# Hydrogen-rich water: a key player in boosting wheat (*Triticum aestivum* L.) seedling growth and drought resilience

**DOI:** 10.1038/s41598-023-49973-7

**Published:** 2023-12-18

**Authors:** Md. Ariful Islam, Most. Nourin Akther Shorna, Shirmin Islam, Suvro Biswas, Jui Biswas, Synthia Islam, Amit Kumar Dutta, Md. Salah Uddin, Shahriar Zaman, Md. Akhtar-E-Ekram, Asad Syed, Ling Shing Wong, Md Sayeedul Islam, Md. Abu Saleh

**Affiliations:** 1 Microbiology Laboratory, Department of Genetic Engineering and Biotechnology, University of Rajshahi, Rajshahi, 6205 Bangladesh; 2 Department of Botany, University of Rajshahi, Rajshahi, 6205 Bangladesh; 3 Department of Agribusiness, Bangabandhu Sheikh Mujibur Rahman Agricultural University, Gazipur, 1706 Bangladesh; 4 Department of Microbiology, University of Rajshahi, Rajshahi, 6205 Bangladesh; 5 Department of Botany and Microbiology, College of Science, King Saud University, P.O. Box 2455, 11451 Riyadh, Saudi Arabia; 6 Faculty of Life and Health Sciences, INTI International University, Putra Nilai, 71800 Nilai, Negeri Sembilan Malaysia; 7 Department of Biological Sciences, Graduate School of Science, Osaka University, Machikaneyama‑Cho 1‑1, Toyonaka, Osaka 560‑0043 Japan

**Keywords:** Biotechnology, Plant sciences

## Abstract

In the modern world, wheat, a vital global cereal and the second most consumed, is vulnerable to climate change impacts. These include erratic rainfall and extreme temperatures, endangering global food security. Research on hydrogen-rich water (HRW) has gained momentum in plant and agricultural sciences due to its diverse functions. This study examined the effects of different HRW treatment durations on wheat, revealing that the 4-h treatment had the highest germination rate, enhancing potential, vigor, and germination indexes. This treatment also boosted relative water content, root and shoot weight, and average lengths. Moreover, the 4-h HRW treatment resulted in the highest chlorophyll and soluble protein concentrations in seeds while reducing cell death. The 4-h and 5-h HRW treatments significantly increased H_2_O_2_ levels, with the highest NO detected in both root and shoot after 4-h HRW exposure. Additionally, HRW-treated seeds exhibited increased Zn and Fe concentrations, along with antioxidant enzyme activities (CAT, SOD, APX) in roots and shoots. These findings suggest that HRW treatment could enhance wheat seed germination, growth, and nutrient absorption, thereby increasing agricultural productivity. Molecular analysis indicated significant upregulation of the Dreb1 gene with a 4-h HRW treatment. Thus, it shows promise in addressing climate change effects on wheat production. Therefore, HRW treatment could be a hopeful strategy for enhancing wheat plant drought tolerance, requiring further investigation (field experiments) to validate its impact on plant growth and drought stress mitigation.

## Introduction

Wheat (*Triticum aestivum* L.) is an essential crop that plays a crucial role in ensuring food security and fueling the global economy through various value chains. Due to the growing human population worldwide and decreasing agricultural land, the need for wheat products is projected to rise by 60% by the year 2050^1^. Therefore, meeting the global demand for wheat will require an annual increase in wheat yields of 1.6%^1^. Wheat contains carbohydrates (71%), protein (13%), water (13%), fat (1.5%), as well as trace amounts of phosphorus, niacin, and dietary fiber^2,3^. However, climate change-induced droughts can negatively affect agriculture-based systems by causing irregular rainfall patterns, requiring adjustments to crop cycles, promoting changes in diseases and insect dynamics, ultimately limiting the potential for increasing wheat production^4^.

In the twenty-first century, food security is facing humanity's most significant challenges, which are the constantly increasing global population and the highly unpredictable climate. The impact of climate change, including extreme temperatures and erratic rainfall patterns, will have consequences on wheat, which is the world's second most widely consumed cereal crop^5^. The growth and productivity of wheat are negatively affected by climate change, as it causes detrimental effects on plant physiological processes, such as crop evapotranspiration (ETc) and water use efficiency (WUE)^6,7^. A decline in global wheat yields of approximately 6.0 ± 2.9% is expected with every 1° increase in global temperature^6^. Additionally, research conducted in Iran indicates that climate change will result in a decrease in grain yield and WUE, accompanied by a higher crop water requirement^5,7^.

At present, predicting drought, a slowly evolving climatic phenomenon with the longest duration, is highly unlikely^8^. Drought can take on different forms such as meteorological, agricultural, hydrological, and socioeconomic depending on factors such as the duration of low rainfall, deficits in soil moisture, or depletion of surface and groundwater storage^9^. Numerous studies have reported that drought conditions have various impacts on agriculture, including a direct effect on the productivity of wheat^10,11^. By the end of the twenty-first century, there is a projection that agricultural regions affected by global drought will experience a significant increase^12^. The production of 75% of the global wheat harvested area was affected by drought, leading to an average reduction of 0.29 t ha^−1^^13,14^.

The condition of drought stress is marked by insufficient water availability, causing various changes in morphology, biochemistry, physiology, and molecular structure^15^. Drought stress affects various aspects of plant functioning such as photosynthesis, chlorophyll production, nutrient metabolism, ion absorption and translocation, respiration, and carbohydrate metabolism^15^. In order to survive under drought conditions, wheat must adapt, and researchers have developed various resistant genotypes that can help maintain levels of soluble sugars, proline content, amino acids, chlorophyll content, as well as enzymatic and non-enzymatic antioxidant activities^16^. Drought stress caused a decline in grain production by 11–34% and a shorter grain filling period by 15–24%^17^. Winter wheat yield decreased by 3.09 t × ha^−1^ (46.8%) in semiarid conditions when compared to irrigated conditions^18^.

Drought stress has a significant impact on wheat cultivation in Bangladesh, a country heavily reliant on wheat as a second staple crop^19^. The most favorable temperature for wheat cultivation is approximately 20 °C, and it lies within the range of 17–23 °C^20^. In Bangladesh, where the lowest recorded temperature hovers at approximately 15 °C and the highest reaches 35 °C, the optimal temperature range for cultivating wheat is between 20 and 25 °C^20^. Moreover, the northwestern region is largely prone to drought conditions and encounters significantly reduced precipitation levels when compared to other areas of the country, suggesting that the temperature in this particular region is also higher than in other parts of the country^21^. Drought stress leads to various consequences such as diminished crop yield, stunted growth, premature ripening, reduced nutritional value, heightened vulnerability to diseases, and financial losses for farmers ^19^.

Plants or plant tissues can receive H_2_ as either a gas or a dissolved solution, which is typically created by bubbling H_2_ through water to create hydrogen-rich water (HRW). HRW can be administered to plants by either adding it to the soil or feed solution or by spraying it onto the foliage^22^. Under stressful conditions such as the presence of excessive metal ions, hydrogen-rich water (HRW) has been shown to promote root growth^23^. The relief of stress by H_2_ is believed to involve phytohormone signalling, as suggested by some studies^22,24^. Sun et al.^25^ assert that H_2_ is safe for human consumption and, as a result, is safe to use in plant treatments for food crops. Wu et al.^26^ found that cabbage showed increased levels of antioxidants when subjected to cadmium stress, while Chen et al.^27^ observed that the antioxidant capacity of *Hypsizygus marmoreus* (a type of mushroom) was elevated through the use of HRW during the postharvest period.

Despite ongoing research, the specific role of HRW in mitigating wheat's susceptibility to drought stress remains uncertain. The main aim of this manuscript was to determine the optimal duration for soaking seeds in HRW (hydrogen-rich water) and to elucidate how HRW contributes to enhancing drought resistance in wheat. This was achieved by examining the morphological and physiological transformations that took place after the seed immersion. The potential identification of increased expression of *Dreb1* genes in wheat, following treatment with HRW, could contribute to enhancing drought stress tolerance. Furthermore, the study included correlation analysis to enhance and support the findings obtained during the research.

## Results

### Morpho-physiological characteristics analysis

**Figure 1:**
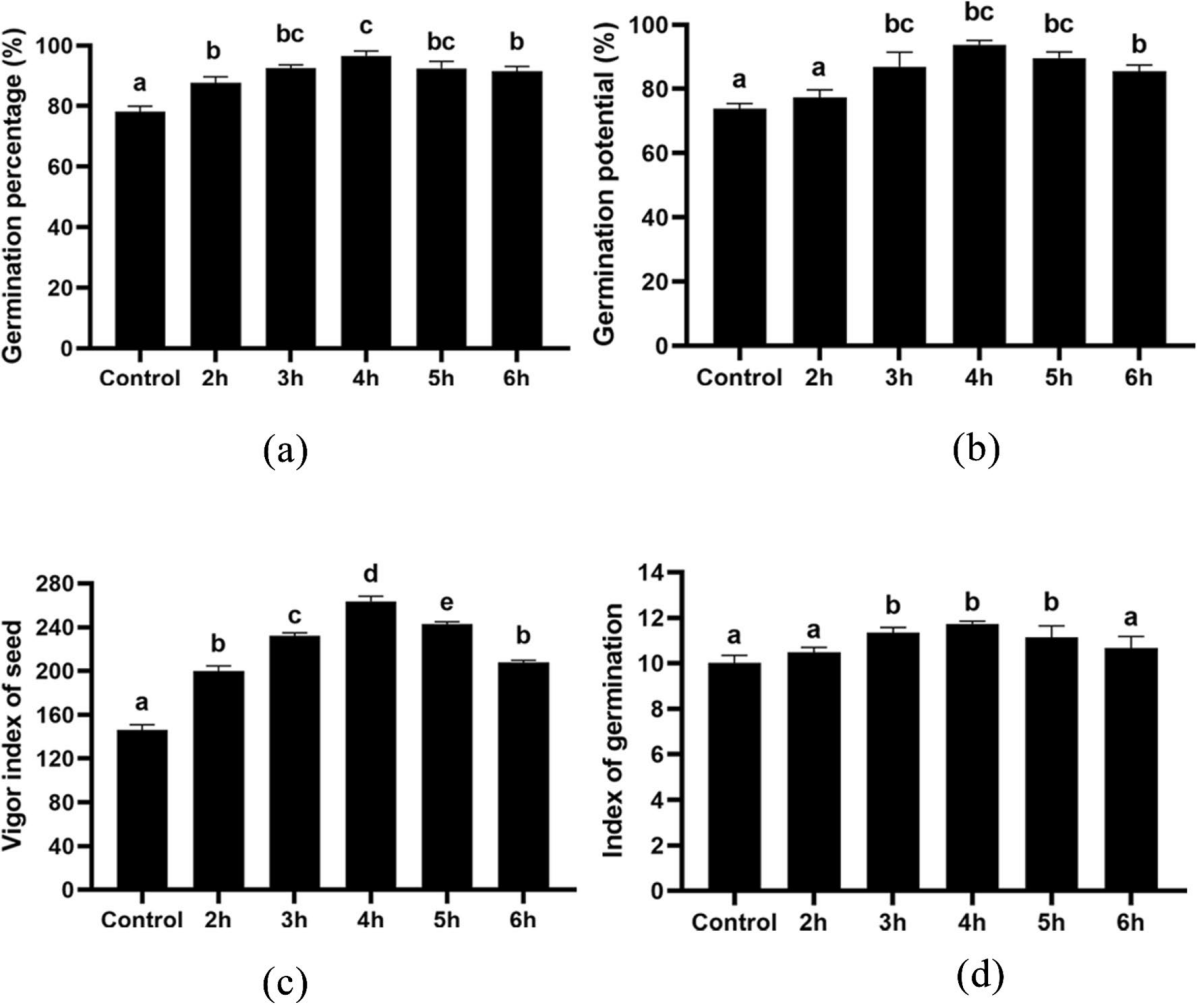
Germination parameters of wheat seeds; (**a**) illustrates germination percentage, (**b**) illustrates germination potential, (**c**) illustrates vigor index of seeds and (**d**) illustrates index of germination. Different letters used to indicate significant differences between the mean ± SD of replications (n = 3) at a significance level of P < 0.05.

**Figure 2:**
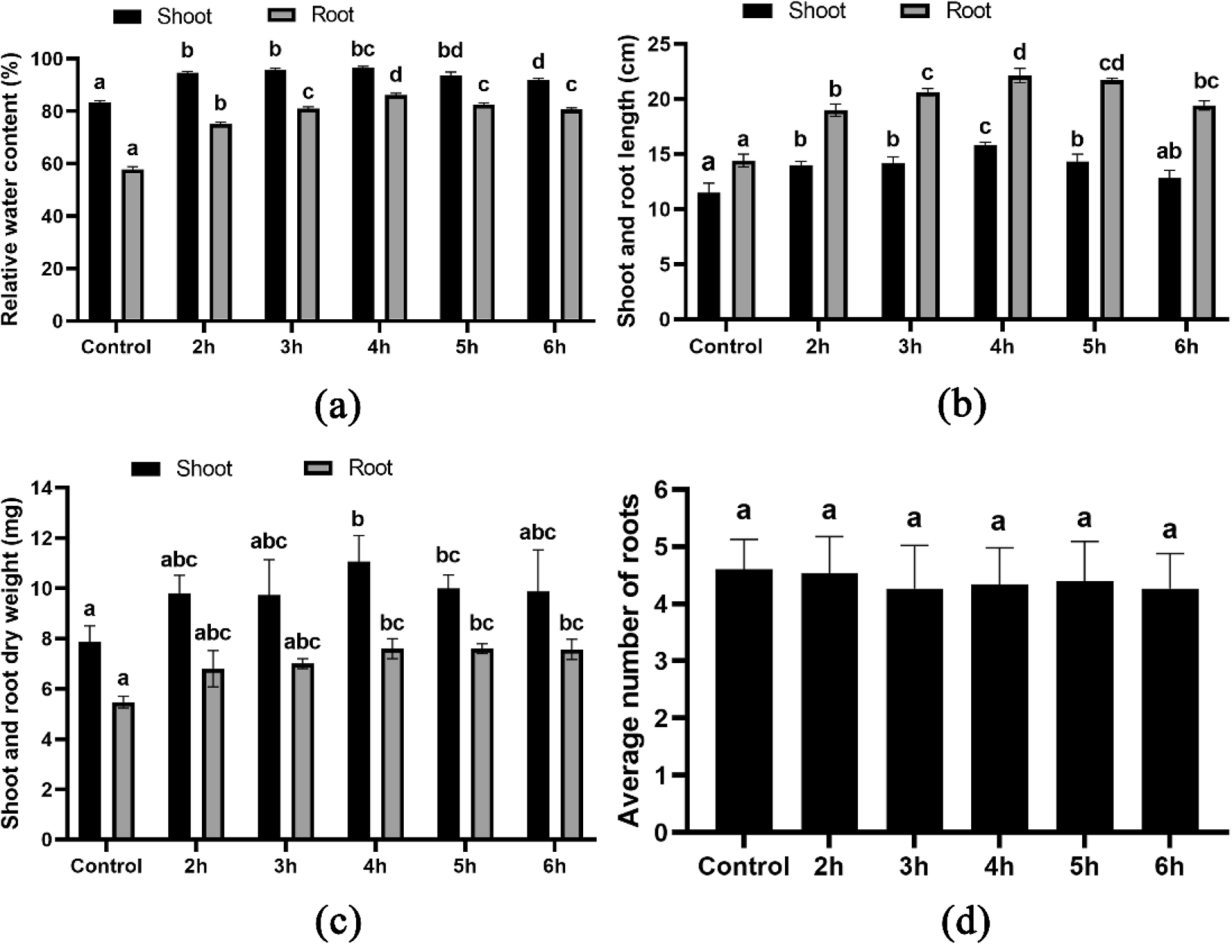
Morphological characteristic of wheat seedlings; (**a**) illustrates relative water content percentage, (**b**) illustrates shoot and root length, (**c**) illustrates shoot and root dry weight and (**d**) illustrates average number of roots. Different letters used to indicate significant differences between the mean ± SD of replications (n = 3) at a significance level of P < 0.05.

The study examined the effect of different durations of treatment with HRW on the germination rate and growth of wheat. The results showed that the 4-h treatment had the highest germination rate of 96.66% (Fig. 1a) and significantly improved germination potential (Fig. 1b), vigor index (Fig. 1c) and germination index (Fig. 1d) compared to the control group. Furthermore, the 4-h treatment led to an increase in relative water content (Fig. 2a), root and shoot dry weight (Fig. 2b) and average root and shoot length (Fig. 2c) compared to the control group. Specifically, after 10 days of treatment, the 4-h HRW treatment resulted in a relative water content in the root of 86.12% and in the shoot of 97.33% (Fig. 2a), an average root length of 22.13 cm (Fig. 2b), and average shoot height of 16.17 cm (Fig. 2b). The average root-shoot dry weight for the 4-h HRW treatment was 7.60 mg and 11.07 g, respectively (Fig. 2c). There was no discernible distinction in the number of roots (Fig. 2d) between the control and treatment groups of plants.

### Chlorophyll and cell death measurement

**Figure 3:**
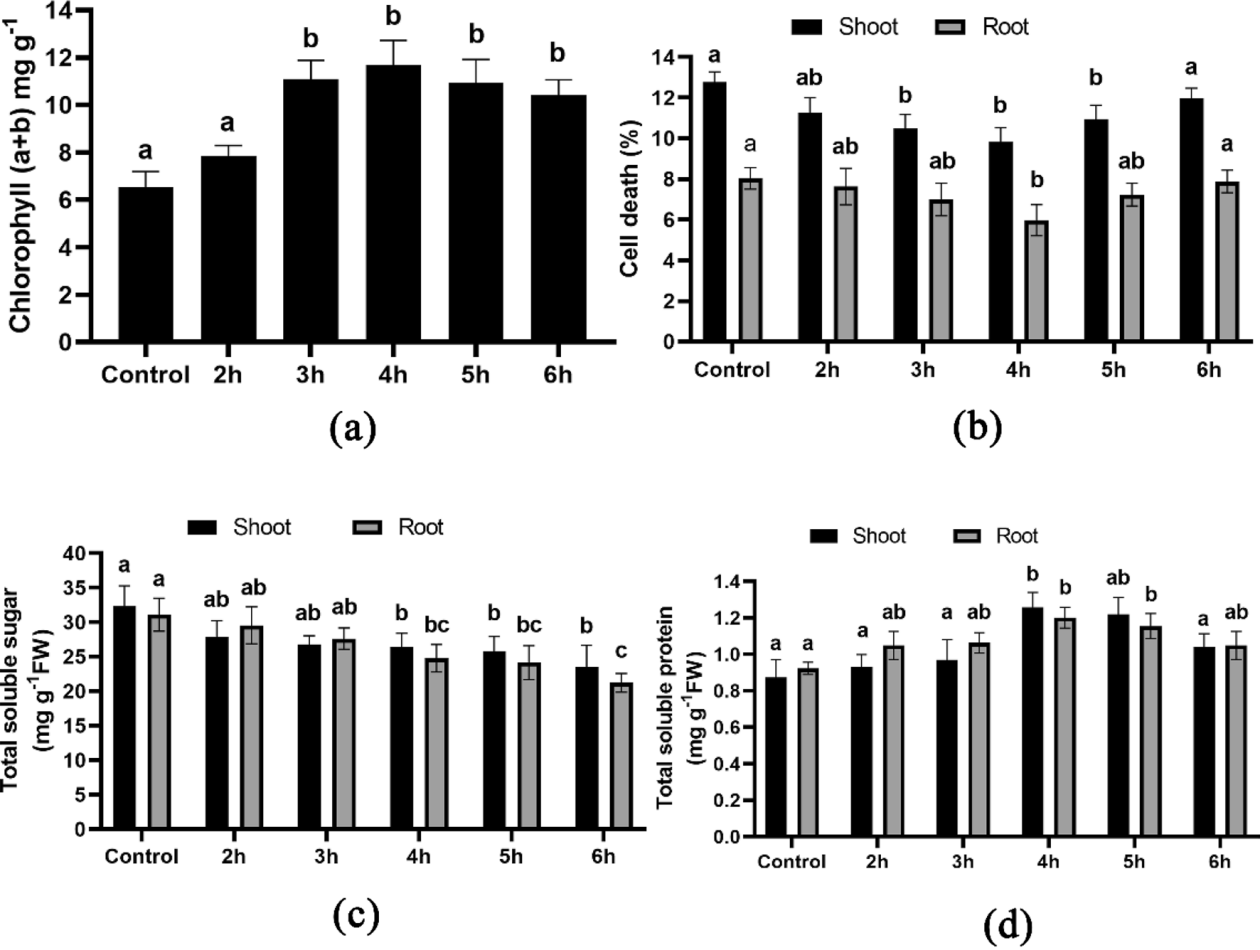
Biochemical characterization of wheat seedlings; (**a**) illustrates chlorophyll (a + b) content, (**b**) illustrates cell death percentage, (**c**) illustrates total soluble sugar and (**d**) illustrates total soluble protein. Different letters used to indicate significant differences between the mean ± SD of replications (n = 3) at a significance level of P < 0.05.

The investigation revealed that all treatments exhibited distinct concentrations of chlorophyll (chlorophyll a and b). The seeds treated with HRW for 4 h resulted in the maximum concentration of chlorophyll, measuring 11.69 mg/g (fresh weight) (Fig. 3a). In comparison to the control group, the seeds treated with HRW exhibited lower percentages of cell death. Furthermore, the root and shoot of plants treated with HRW for 4 h exhibited the lowest percentage of cell death, with values of 5.98% and 9.82% (Fig. 3b), respectively. Conversely, the control group recorded higher cell death percentages, with values of 8.02% and 12.76% (Fig. 3b) for root and shoot, respectively.

### The impact on the concentration of total soluble sugar and protein

The study found that the total soluble sugar content gradually decreased in the plants treated with different durations of HRW compared to the control plants. The 6-h HRW treatment resulted in the lowest sugar concentration in both the root and shoot, with values of 21.24 mg g^−1^ and 23.51 mg g^−1^ (Fig. 3c) respectively, as compared to the control values of 31.10 mg g^−1^ and 32.36 mg g^−1^ (Fig. 3c). Conversely, the highest total soluble protein was observed in the 4-h treatment plants, with values of 1.16 mg g^−1^ and 1.29 mg g^−1^(Fig. 3d) in the root and shoot, respectively, as compared to the control values of 0.92 mg g^−1^ and 0.87 mg g^−1^(Fig. 3d).

### H_2_O_2_ and NO concentration measurement

**Figure 4:**
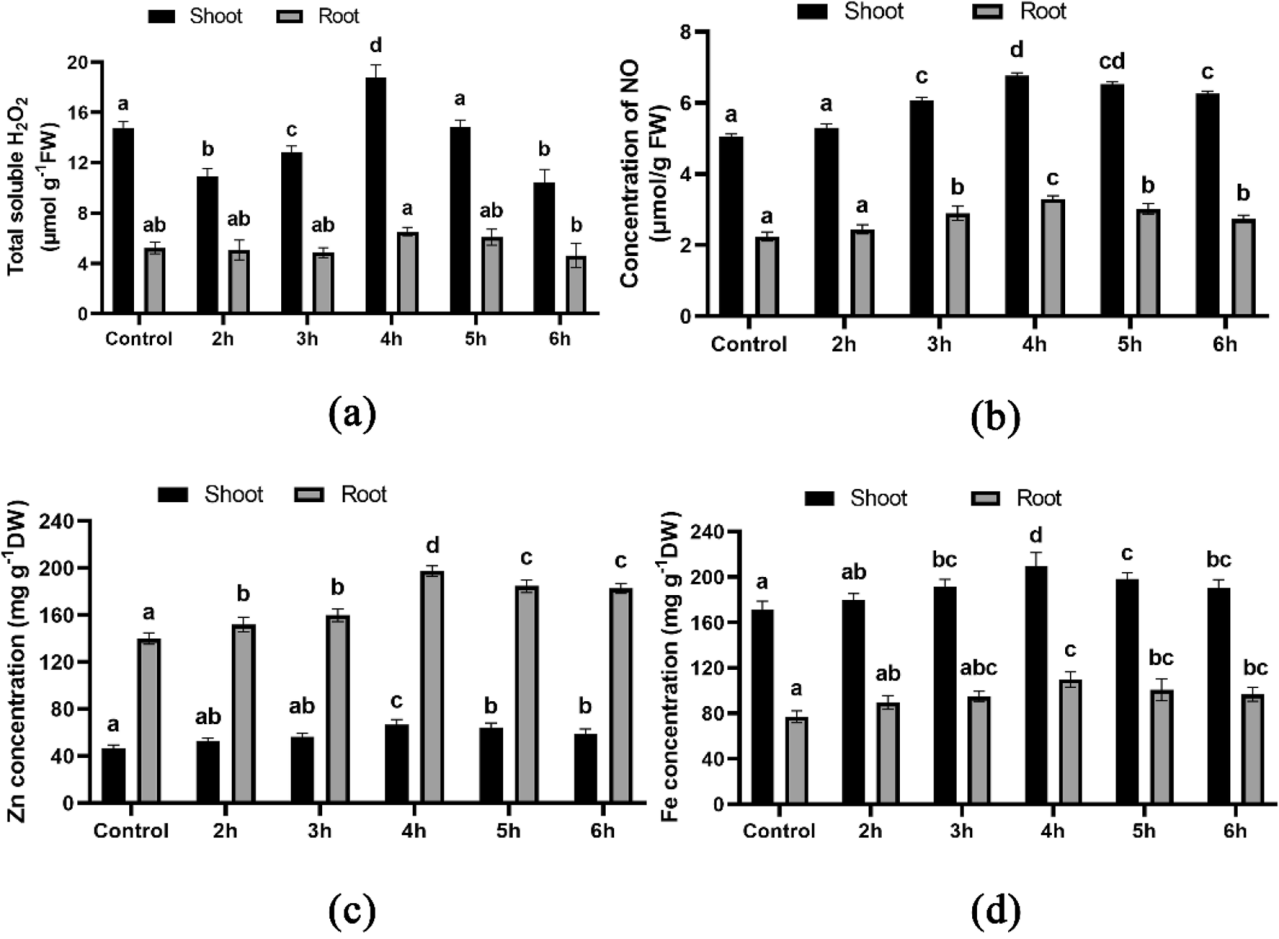
Biochemical characterization of wheat seedlings; (**a**) illustrates total soluble H_2_O_2_, (**b**) illustrates NO concentration, (**c**) illustrates Zn concentration and (**d**) illustrates Fe concentration. Different letters used to indicate significant differences between the mean ± SD of replications (n = 3) at a significance level of P < 0.05.

In the 4-h and 5-h treated seeds with HRW, there was a notable increase in the concentration of H_2_O_2_ as compared to the control group. The highest concentration of H_2_O_2_, measuring 6.55 μmol g^−1^ FW (Fig. 4a), was found in the root, while the shoot had a concentration of 18.78 μmol g^−1^ FW (Fig. 4a). However, the other treated seed groups showed a slight decrease in H_2_O_2_ concentration compared to the control group. The concentration of NO showed significant changes in both the root and shoot of the plants treated with HRW, as compared to the control group. The highest amount of NO was detected in both the root and shoot of the plants after being exposed to HRW treatment for 4 h, with a recorded measurement of 3.30 μmol g^−1^ FW (Fig. 4b) and 6.77 μmol g^−1^ FW (Fig. 4b), respectively, as compared to the control root and shoot, which measured 2.24 μmol g^−1^ FW (Fig. 4b) and 5.06 μmol g^−1^ FW (Fig. 4b), respectively.

### Zn and Fe concentration measurement

Compared to the control group, the seeds treated with HRW exhibited a significant increase in Zn and Fe concentrations in both root and shoot. The root and shoot of the 4-h HRW-treated plants had the highest observed Zn concentration of 197.43 mg g^−1^ DW (Fig. 4c) and 66.98 mg g^−1^ DW (Fig. 4c), respectively, while the control group recorded 140.06 mg g^−1^ DW in root (Fig. 4c) and 46.23 mg g^−1^ DW in shoot (Fig. 4c). Similarly, the maximum Fe concentration of 109.83 mg g^−1^ DW in root (Fig. 4d) and 209.98 mg g^−1^ DW in shoot (Fig. 4d) was observed in the 4-h HRW-treated group, while the control group had 71.11 mg g^−1^ DW in root (Fig. 4d) and 171.56 mg g^−1^ DW in shoot (Fig. 4d).

### The impact of antioxidant enzymes (SOD, APX, CAT) on young plants

**Figure 5:**
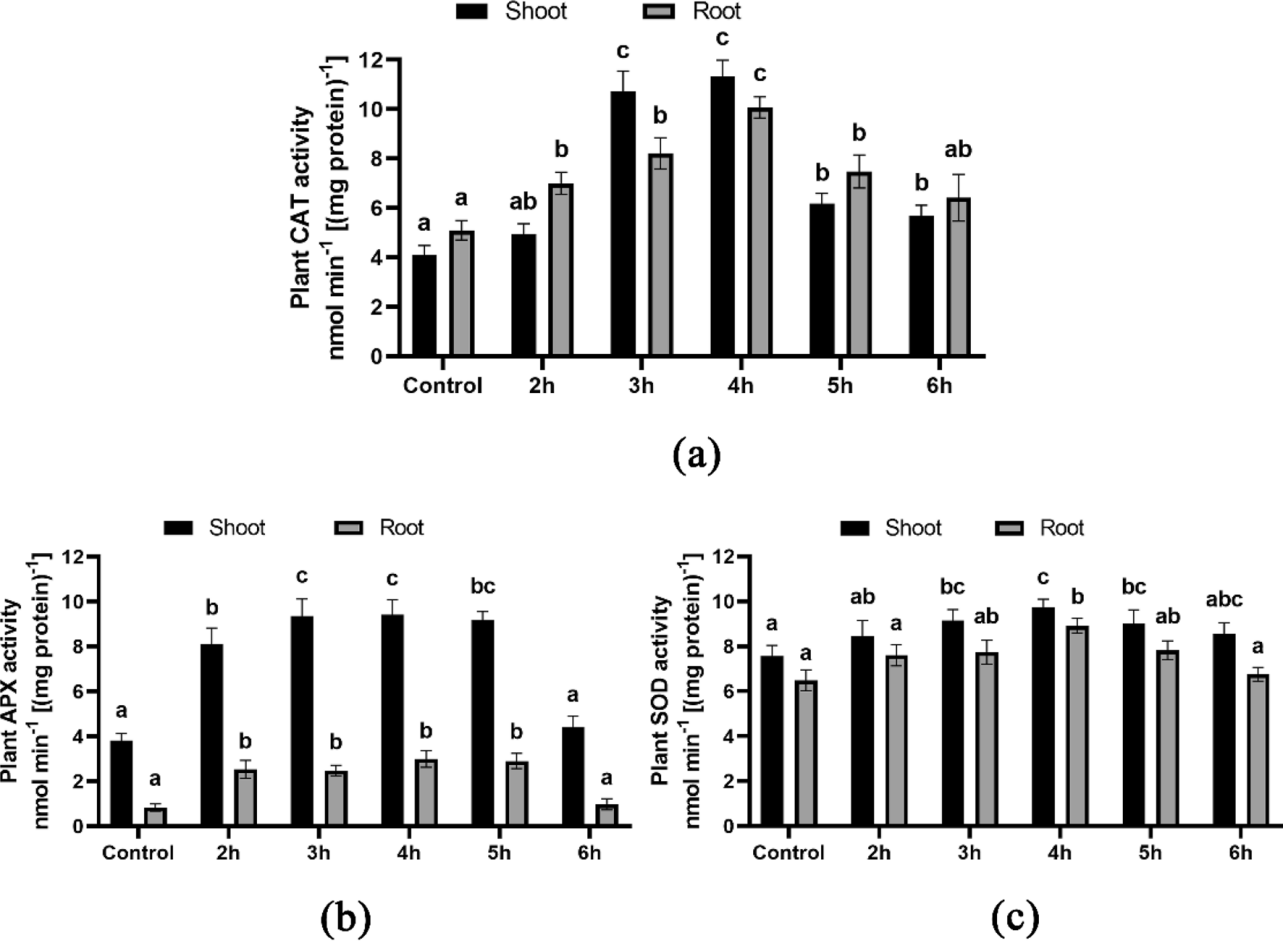
Antioxidant activity of wheat seedlings’ shoot and root; (**a**) illustrates CAT activity, (**b**) illustrates APX activity and (**c**) illustrates SOD activity. Different letters used to indicate significant differences between the mean ± SD of replications (n = 3) at a significance level of P < 0.05.

The levels of antioxidant activity were notably higher in the 3-h, 4-h, and 5-h HRW treatments, but only slightly increased in the other treatments compared to the control. The maximum CAT activity was observed in the root and shoot during the 4-h HRW treatment, with a value of 10.07 nmol min^−1^(mg protein^−1^) (Fig. 5a), and with a value of 11.32 nmol min^−1^ (mg protein^−1^) (Fig. 5a), respectively. The HRW-treated seeds demonstrated increased APX activities in both the shoot and root compared to the control. The 4-h HRW treatment resulted in the highest APX activity levels in the shoot and root, with values of 9.84 nmol min^−1^ (mg protein^−1^) (Fig. 5b) and 2.89 nmol min^−1^ (mg protein^−1^) (Fig. 5b), respectively. Similar results were also observed in the SOD activities, with the maximum activity levels in the shoot and root measured at 9.75 nmol min^−1^ (mg protein^−1^) (Fig. 5c) and 8.92 nmol min^−1^ (mg protein^−1^) (Fig. 5c), respectively.

### Gene expression analysis

**Figure 6:**
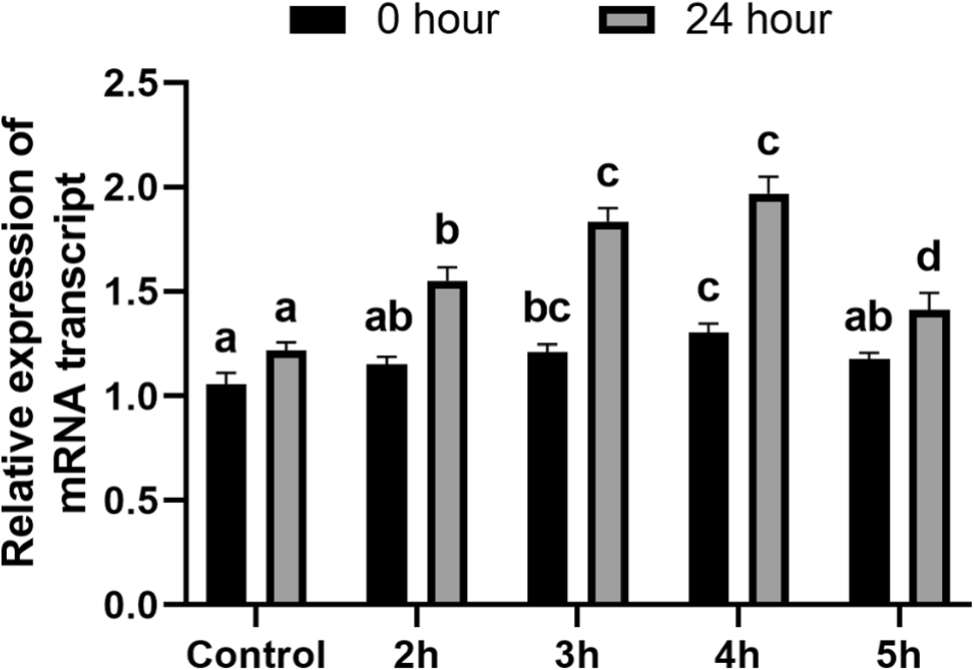
Illustrates relative expression of mRNA transcript of wheat seedlings. Different letters used to indicate significant differences between the mean ± SD of replications (n = 3) at a significance level of P < 0.05.

According to the real-time PCR analysis, the expression of the *Dreb1* gene was relatively consistent across the control, 2 h, 3 h, 4 h, and 5 h HRW treatment groups at the onset of drought induction (0 h). However, after 24 h post-induction of drought, the HRW treated group exhibited a significant increase in *Dreb1* gene expression compared to the control group (Fig. 6). The HRW treatment for 3 h and 4 h demonstrated significantly elevated expression of the *Dreb1* gene compared to both the control group and other treatment groups. Notably, the 4-h HRW treatment exhibited the highest expression of the *Dreb1* gene (Fig. 6), indicating an approximate 90% increase compared to the control group.

### Correlation analysis

**Figure 7:**
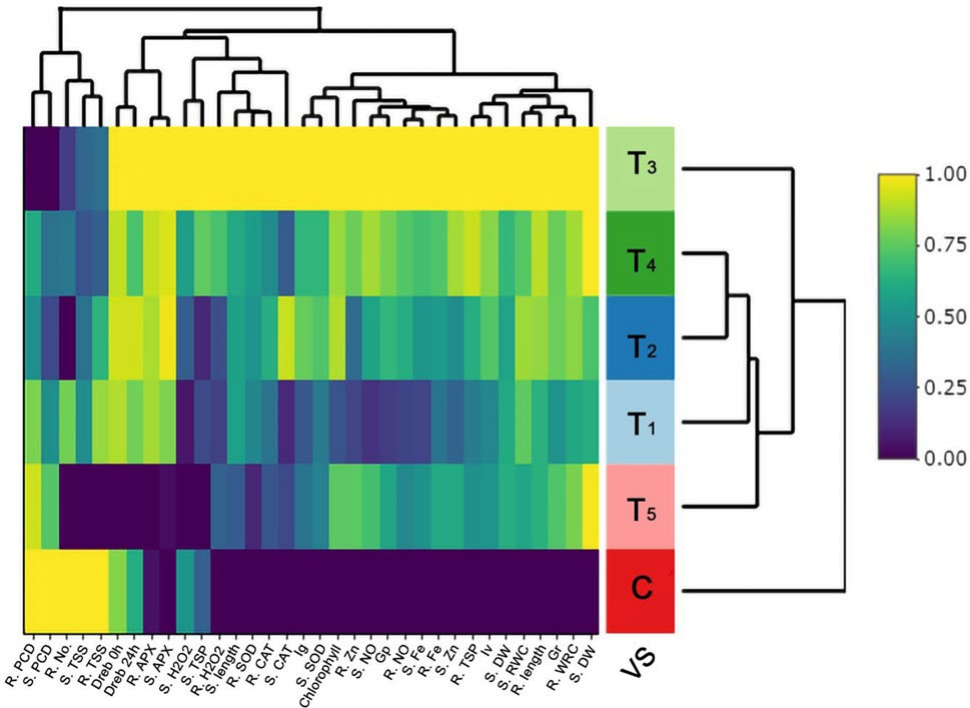
Heat map depicts the significant Pearson correlation among wheat seedlings morphological, biochemical and enzymatically 34 parameters after HRW treated seeds at different time intervals. The yellow color key indicates higher R value, while blue indicates lower value. The seedling parameters S. DW (Shoot dry weight), R. RWC (root relative water content), Gr (germination rate), R. length (root length), S. RWC (shoot relative water content), S. DW (shoot dry weight), Iv (vigor index), R. TSP (total soluble protein of root), S. Zn (Zn concentration of shoot), R. Fe (Fe concentration of root), S. Fe (Fe concentration of shoot), R. NO (NO concentration of root), Gp (germination potential), S. NO (NO concentration of shoot), R. Zn (Zn concentration of root), Chlorophyll (chlorophyll a and b), S. SOD (superoxide dismutase of shoot), Ig (Index of germination), S. CAT (catalase activity of shoot), R. CAT (catalase activity of root), R. SOD (superoxide dismutase activity of root), S. length (shoot length), S. TSP (total soluble protein of shoot), R. H_2_O_2_ (H_2_O_2_ concentration of root), S. APX (ascorbate peroxidase activity of shoot), R. APX (ascorbate peroxidase activity of root), *Dreb1* 24 h (*Dreb1* gene expression after 24 h of treatment), *Dreb1* 0 h (*Dreb1* gene expression after 0 h of treatment), R. TSS (total soluble sugar of root), S. TSS (total soluble sugar of root), R. No. (average root number), S. PCD (program cell death of shoot) and R. PCD (program cell death of root) are mentioned here. In the figure, 4 h treated (T_3_) wheat seed showed highest significant R value (R = 1) compare to rest of the treated seed. The root dry weight was also increased when seeds were treated at 5 h and 6 h likely 4 h treated seeds.

The heat-map (Fig. 7) illustrated the significant Pearson correlation among 34 plants’ morphological, biochemical, and enzymatic parameters after HRW treatment at different time intervals. The yellow color in the heat-map indicated a highly significant R value (R = 1), while the purple color represents a non-significant R value (R = 0). The correlation analysis revealed that the 4-h HRW treatment exhibited a highly significant relationship compared to both the control group and other treatment groups (Fig. 7). However, four parameters, namely root PCD, shoot PCD, root TSS, and shoot TSS, showed non-significance at the 4-h treatment group, which also holds importance for wheat drought tolerance activity. The findings from the Global analysis of similarities (ANOSIM) revealed considerable dissimilarities between the wheat seedlings treated with HRW at different time intervals and the control group (Table S1). The statistical significance (P = 0.001) indicated that these differences are highly significant from a statistical standpoint. During the PCO plot analysis, PCO1 accounted for 47.8% of the total variation, while PCO2 contributed to 27.2% of the total variation (Fig. S1).

## Discussion

Drought is a significant abiotic factor that hinders plant growth and development, which negatively affects agricultural need^28,29^. Half a century ago, researchers found out that introducing external H_2_ gas significantly enhances the process of seed germination^30^. Our experiment revealed that the 4-h treatment of seeds with HRW was significantly more effective than both the control group and other treatment groups in promoting morphological growth, as evidenced by increased germination rate, germination potential, vigor index, root-shoot length and number, as well as fresh and dry weight of root and shoot. After the implementation of HRW treatment to *T. aestivum* L. seeds, notable refinements were picked out in their biochemical profiles, functions, and physical structure. The substantial functional heterogeneity between the seeds subjected to HRW treatment and the control group were evident throughout the entire experimental period. Previous studies have noted a comparable morphological advancement in barley where subjecting HRW to drought circumstances resulted in an increase in the rate of seed germination and a reduction in the rate of inhibition of seedling growth^31^. Furthermore, the treatment of rice plants with HRW resulted in an increase in both plant height and dry weight^32^. Wheat seedlings, altering their physical traits to manage drought stress, frequently employ diverse strategies to confront limited water availability^33–35^. Yet, hydrogen-rich water might have the potential to aid in drought resilience by enabling these alterations in form and structure.

In our present investigation, it was discovered that plants derived from seeds treated with HRW exhibited a significant increase in the accumulation of both RWC and chlorophyll content, both of which are crucial for plant growth, with RWC playing a crucial role in maintaining water status and stress tolerance^29^, while chlorophyll is an essential component for photosynthesis and energy production^36^. Prior several studies have indicated that administering HRW can mitigate the reduction in chlorophyll content caused by salt stress in both cucumber seedlings^37^ and *Arabidopsis*^38^. In comparison to the distilled water control group, the application of HRW resulted in a gradual reduction of soluble sugar content in both root and shoot of the plant. Specifically, the total soluble sugar content showed a significant decrease in the group treated with HRW for 6 h, in contrast to other treatment groups. A previous study reported that decreasing the level of soluble sugar in plants enhances their ability to withstand environmental stressors like drought and heat^39^. Conversely, a significant rise in soluble protein content was noticed, with seeds treated with HRW for 4 h exhibiting maximum values of 1.15 mg/g and 1.29 mg/g, in contrast to the control, for the four respective treatment condition. Previous research has found that HRW enhances total soluble protein levels in cucumber seedlings to mitigate salt tolerance ^37^ and in alfalfa to alleviate cadmium toxicity^40^. Research exploring the effects of hydrogen-rich water on wheat seedlings’ physiology suggests its potential to mitigate the effects of drought stress by modulating these physiological parameters.

Our research has demonstrated that HRW treatment for a duration of 4 h significantly increased the levels of H_2_O_2_ and NO in both the root and shoot of wheat seeds, as compared to the control group. This finding is noteworthy as H_2_O_2_ and NO are pivotal signaling molecules that participate in a wide range of developmental and physiological processes in plants, including germination, growth, root organogenesis, pollen tube growth, flowering, and responses to biotic and abiotic stimuli^31,41^. Our findings also suggest that the application of HRW treatment to wheat seeds, particularly for 4 h, can markedly increase the concentrations of Zn and Fe in both the root and shoot, thus indicating the promising potential of HRW treatment for enhancing the levels of these nutrients in plants. Several studies have reported that the provision of sufficient quantities of Zn and Fe nanoparticles can alter the drought tolerance of crops such as wheat, sunflower, tomato, and red cabbage ^41,42^. H_2_O_2_, NO, Fe, and Zn are vital for boosting wheat plants' resilience to drought as well as maintaining their balanced levels is crucial for optimal plant function^42^. Regulating these elements through hydrogen-rich water treatment could greatly enhance wheat seedlings' ability to withstand drought stress^31^.

In arid environment, plant cells generate operational reactive oxygen species (ROS), while their antioxidant enzymes function as inhibitors of ROS generation and reactivity to environmental stresses. These enzymes, such as superoxide dismutase (SOD), ascorbate peroxidase (APX), and catalase (CAT), are crucial in mitigating oxidative damage and reducing ROS production in plants. Among them, CAT is particularly noteworthy in scavenging H_2_O_2_ to impede ROS formation^32,37,43^. Our study revealed that the application of HRW to plants led to enhanced levels of CAT, SOD, and APX activities relative to the control group. In this study wheat seedlings that were exposed to HRW for 4–5 h exhibited a noteworthy boost in the activities of these enzymes relative to other treatment groups. Furthermore, the enzymes ascorbate peroxidase (APX), catalase (CAT), and superoxide dismutase (SOD) play critical role in enhancing drought tolerance in wheat plants^33,44^.

A recent study found that certain genes, such as *DREB1*^45^, *TaMYB30-B*^46^, *TaASR1*^47^, *AtWRKY30*^48^ and *HVA1*^49^ greatly enhance wheat's resistance to drought stress. To demonstrate the expression of wheat genes related to drought stress, we examined the expression of *DREB1* genes following drought induction. Our results indicated that HRW has a notable ability to naturally induce the expression of the *DREB1* gene. Remarkably, the 4-h treatment of HRW showed the highest expression of the *DREB1* gene, signifying an approximate 90% increase compared to the control group. This finding strongly suggests that HRW has the potential to enhance the drought tolerance of wheat.

The application of HRW treatment on wheat seeds led to substantial enhancements in growth factors and the accumulation of essential components in wheat plants. It elevated nutrient levels and boosted antioxidant enzyme activity, thereby mitigating oxidative damage and reducing ROS production. HRW exhibited potential in devising novel approaches to combat the detrimental impacts of climate change on wheat production, including the activation of drought tolerance genes.

## Methods

### Preparation of hydrogen-rich water (HRW)

In this experiment, utilized a hydrogen water generator to produce hydrogen-rich water (HRW), which is water that contains dissolved molecular hydrogen gas. The generator employed an electrolysis process that split the water molecules into hydrogen gas and oxygen gas. The hydrogen gas was then dissolved into the water, creating hydrogen-rich water. The hydrogen water generator also included a mechanism to separate the hydrogen and oxygen gases, ensuring that only hydrogen gas was dissolved into the water. The hydrogen water generator utilized in the experiment has a cylindrical shape and features an output dispenser for dispensing the hydrogen-rich water. The generator's body is constructed using e glass or metal, while the electrodes are composed of platinum and titanium. This particular hydrogen machine has a capacity of 380 mL, and through the process of electrolysis, it can produce high hydrogen concentrations of up to 1500 ppb.

### Seed treatment with HRW

HRW was prepared by hydrogen water generator. Then wheat seeds (BARI-33) were washed thoroughly and immersed in distilled water for 30 min that are collected from Regional Wheat Research Institution, Rajshahi. We followed established protocols for collecting plant specimen, ensuring that our actions do not endanger the survival of plant species. The plant materials and data were used in a responsible and ethical manner. Distilled water was used for control and HRW was used for treatment seeds. Randomly selected wheat seeds were submersed in six different petri dishes as control, 2-h, 3-h, 4-h, 5-h and 6 h. After the treatment the control and treated wheat seeds were placed into each 90 mm petri dish containing 2 layers moistened of tissue papers at the bottom for germination. A total of 720 (3 replicates × 6 groups × 40 seeds) wheat seeds were selected randomly and divided into six groups as per instruction of ISTA 2018 [International Seed Testing Association (ISTA)]. Then the petri dishes were taken at growth chamber at 23 ± 2 °C temperature and 50% humidity for germination parameters observation.

### Morpho-physiological characteristics

The germination parameters and plant growth were evaluated according to the procedure described by Zhou et al.^50^. The germination rate (GR), germination potential (GP), Index of Germination (IG), and Vigor Index (VI) were calculated using the same method as previously reported. After 10 days in the petri dishes, the plant growth was assessed. The plants were carefully removed from the tissue paper, and their roots were washed with tap water. The lengths of the roots and shoots were measured, and the number of roots was recorded.

#### Assessing the relative water content (RWC)

The method for determining the relative water content of plant roots and shoots, as described by González and González-Vilar^51^, involved taking five plants from petri dishes, washing the roots and removing any excess water, and measuring the fresh weight (FW) of the root and shoot separately. The roots and shoots were immersed in petri dishes containing distilled water for a duration of 2 h, and their turgid weight (TW) was recorded. Next, the dry weights (DW) of plant roots and shoots were determined following the method described by Arshadullar and Zaidi S^52^. The plant shoots were cut at their base and allowed to dry for 4 days at a temperature of 75 °C to eliminate moisture. Once completely dried, the weight of the shoots was measured using an electronic scale. Likewise, the roots from the same plants underwent a 4-days incubation at 75 °C for moisture removal and were subsequently weighed using an electronic scale. The RWC was then calculated using a formula by WEATHERLEY^53^.

$$\mathrm{RWC }= \sum \frac{{\text{FW}}-{\text{DW}}}{{\text{TW}}-{\text{DW}}} \times 100$$

### Biochemical changes analysis

#### Chlorophyll (a + b) determination

To determine the chlorophyll concentration, fresh young shoots were ground with a mortar and pestle in 90% methanol. The mixture was then centrifuged, and the supernatant was collected in tubes. The optical density of the supernatant was measured at 662 nm for chlorophyll a and 653 nm for chlorophyll b using a spectrometer (Analytic Gena, Germany). The total chlorophyll concentration was calculated using a standard procedure^54^.

#### Assessing the concentration of total soluble sugar

To determine the total soluble sugar concentration in the roots and shoots, the method described by Dubois et al.^55^ was used. The roots and shoots were homogenized in aqueous ethanol (v/v 80%) at 12,000 rpm for 5 min, and the resulting supernatants were collected in tubes. Next, 0.2% anthrone reagent was mixed with the clear supernatant. The sample mixtures were incubated in a boiling water bath for 8 min and immediately placed on ice. In order to ascertain the overall concentration of soluble sugars, the ice-cold samples' optical density was assessed at 620 nm. Subsequently, the total soluble sugar content was quantified by comparing the optical density data at 620 nm with a standard glucose curve.

#### Assessing the concentration of total soluble protein

The amount of soluble protein in both roots and shoots was determined using a spectrophotometric method^56^. To isolate the protein, the roots and shoots were thoroughly washed, weighed, and finely ground using a chilled mortar and pestle in a buffer solution comprising 2 mM EDTA, 50 mM Tris–HCl, pH 7.5, and 0.04% (v/v) 2-mercaptoethanol. The resulting homogenates were subsequently subjected to centrifugation at 12,000 rpm for 10 min at 25 °C, and the clear liquid (supernatant) was then transferred into quartz cuvettes. The clear liquid obtained after centrifugation was combined with 1 mL of Coomassie Brilliant Blue (CBB), and its optical density was measured at 595 nm using a spectrophotometer (Analytic Gena, Germany). The total soluble protein content was determined by comparing the optical density data at 595 nm with a standard curve of BSA (bovine serum albumin)^56^.

### Assessing the concentration of nitric oxide (NO)

The level of NO in seedlings’ roots, and shoots was measured by monitoring the changes in the absorption of hemoglobin, which results in the conversion of oxyhemoglobin (HbO_2_) to methemoglobin (metHb) in the presence of NO^57^. The samples were homogenized in a chilled NO buffer (1 mL) and then centrifuged at 10,000 rpm for 10 min. The resulting supernatants were combined with a 5 mM HbO_2_ solution and left to incubate for 7 min at room temperature. To assess the conversion rate of HbO_2_ to methemoglobin (metHb), which serves as an indicator of the presence of NO, the optical density was measured at 401 nm using spectrophotometer (Analytic Gena, Germany).

### Assessing the concentration of H_2_O_2_

The study by Alexieva et al.^58^, involved measuring H_2_O_2_ levels in fresh roots and shoots. The procedure comprised of rinsing the samples with distilled water, blending them in 0.1% TCA, and then subjecting the homogenate to centrifugation. The resulting supernatant was combined with 1 M KI and 10 mM phosphate buffer, left in darkness for an hour, and afterward, the optical density of the obtained extract was measured at 390 nm using a spectrophotometer.

### Assessing the cell death

To analyze cell death in both the roots and shoots, the Evans blue method^59^ was used with some modifications. Initially, the separated root and shoot samples were immersed in a 0.25% Evans blue solution at room temperature for 15 min. Next, the solution was replaced with 1.0 mL of 80% ethyl alcohol and incubated for 10 min. The samples were then placed in a water bath at 50 °C for 15 min, followed by centrifugation at 12,000 rpm for 10 min. The clear supernatant was subjected to spectrophotometric analysis at 600 nm, and the occurrence of cell death was evaluated by comparing it to the absorbance of untreated fresh root and shoot samples.

### Assessing the Zn and Fe concentration

In accordance with Kabir et al.^60^, the concentration of Zn an Fe were determined. Initially, plant tissues (both root and shoot) were collected and washed with 1 mM CaSO_4_ for 5 min followed by a thorough rinse with distilled water. The cleaned samples were then dried in an incubator at 80 °C for 4  days. After the drying process was completed, the plant tissues were mixed with 3 mL HNO_3_ and 1 mL H_2_O_2_ in a test tube and heated at 75 °C for 12 min. The concentrations of Zn and Fe were examined through Flame Atomic Absorption Spectroscopy, employing an ASC-6100 auto sampler and an air-acetylene atomization gas mixture system (Model No. AA-6800, Shimadzu). Standard solutions of Zn and Fe were individually prepared to establish their respective concentrations.

### Determining the concentration of antioxidant enzymes (CAT, SOD and APX)

The CAT, SOD and APX enzymes activity of the plant root and shoot were measured by following the method. To measure all antioxidant activities, 0.3 g of leaf and root samples were separately crushed in phosphate buffer (10 mL, 100 mM, pH 7.0) and then the resulting homogenate samples were centrifuged at 12,000 rpm for 12 min to separate the supernatant. For CAT activity, a reaction mixture of 2 mL was prepared with potassium phosphate buffer (1.5 mL, 100 mM, pH 7.0), H_2_O_2_ (400 μL of 6%), and 100 μL of leaf extract. The reduction in absorbance was assessed at 240 nm using a UV–Vis spectrophotometer (T60 UV Visible Spectrophotometer), and the measurement of CAT activities was carried out following the method described by Verma and Dubey^61^. For SOD, reaction components were prepared by mixing sodium bicarbonate or carbonate buffer (1.3 mL, 50 mM, pH 9.8), EDTA (100 µL, 0.1 mM), and Epinephrine (500 µL, 0.6 mM). Then, adrenochrome formation was measured at 475 nm (T60 UV Visible Spectrophotometer). Subsequently, the SOD activity of the samples is determined by computing the SOD activity based on the acquired absorbance values and then comparing these values to the standard curve created using the known SOD enzyme, as outlined by Sun and Zigman^62^. In the case of APX, reaction mixtures were prepared using potassium phosphate buffer (1 mL of 100 mM, pH 7.0), ascorbic acid (500 μL of 0.2 mM), EDTA (100 μL of 0.2 mM), H_2_O_2_ (300 μL of 6%), and 100 μL of plant extract. The decrease in absorbance was recorded at 290 nm using a UV–Vis spectrophotometer (T60 UV Visible Spectrophotometer) at 10-s intervals up to 1 min and the measurement of APX activities was carried out following the method described by Verma and Dubey^61^ The specific activity of the enzyme was expressed as μmol min^−1^ [(mg protein)^−1^].

### Drought mitigating activity

#### Morphological stress observation

To evaluate plant response to drought stress, ten plants were chosen for each treatment, replicated three times. Watering was withheld for 10 days to simulate stress conditions in potted plants. Following stress induction, discussed below, plants were rehydrated and allowed a recovery period of 5 days. Surviving plants were recorded as rescued individuals.

#### PEG treatment

Polyethylene glycol (PEG), with a molecular weight of 6000, was utilized to artificially impose drought stress on wheat plants^63,64^. Both control and treatment wheat seedlings were subjected to a 15% concentration of PEG, inducing severe drought effects after 10 days of germination. Each Petri dish was supplied with 100 mL of PEG solution and the duration of drought stress endured for 12 days in each treatment. The initial measurements were taken immediately after administering the PEG treatment, with the final readings being collected after 24 h of exposure to PEG.

#### RNA isolation and gene expression analysis

In this study, the methodology described by Rahman et al.^65^ was employed to analyze RNA isolation and gene expression. Specifically, quantitative reverse transcription PCR techniques were used to investigate the expression of *Dreb1* in leaves. To begin, 30 mg of leaves were ground in liquid nitrogen with a mortar and pestle, and total RNA was extracted using the Promega SV total RNA isolation system. Following DNase treatment, 1 μL of extracted RNA from select samples was evaluated for RNA degradation by running it in a 1% agarose gel. Next, first-strand cDNA was generated using the Promega Reverse Transcription systems, and RNA extraction was confirmed by observing it in an agarose gel electrophoresis and quantified by Nano drop. RNase was then utilized to purify the cDNA, and real-time PCR analysis was conducted using the CFX Opus 96 real-time PCR system, with a program consisting of a 3-min incubation at 95 °C, 40 cycles of 30 s at 94 °C, 15 s at 56 °C, and 30 s at 72 °C. Finally, actin was utilized as an internal control to normalize the gene expression levels.

### Statistical analysis

The figures in this study were created using Graph Pad Prism 9.5.1.733. The experimental design involved a one-factor completely randomized approach with three replicates, and data variability and result validity were assessed through two-way analysis of variance using Graph Pad Prism 9.5.1.733 as well. The relationship between parameters was evaluated using correlation analysis and principal component analysis. To compare the means of the various treatments, Turkey's multiple range test was employed with a significance level of 0.05. The data were normalize using square root transformation before statistical analysis. The ANOSIM (Analysis of Similarity) were conducted to identify the significant differences of characters with controls in Primer E (Version 7). The Principal Coordinates Analysis (PCO) showed the ANOSIM based ordination of controls and treatments (2 h, 3 h, 4 h, 5 h and 6 h). Later the pair-wise comparisons were also conducted using Draftsman plots. Finally, Heat map analysis was performed in R (version 4.1.3) using package heatmaply to visualize the Pearson correlation among the 34 parameters. The data were also normalized before analysis in R.

## Statement on experimental research and field studies on plants or plant parts

We acknowledge the intrinsic value of plant life and recognize the importance of preserving genetic diversity and ecological functions. In conducting experimental research on plants or plant parts, we strived to minimize harm to plants and minimize our impact on plant populations and ecosystems. We followed established protocols for collecting plant specimen, ensuring that our actions do not endanger the survival of plant species. The plant materials and data were used in a responsible and ethical manner.

## Statement regarding seed collection

We hereby confirm that the collection of wheat seeds from the Regional Wheat Research Institution in Rajshahi was conducted in strict accordance with the institution's guidelines and regulations. Prior to collecting the seeds, all necessary permissions were obtained from the appropriate authorities at the institution. The collection process adhered to the stipulated protocols to ensure the preservation of the institution's research resources and the maintenance of ethical standards. We express our gratitude to the Regional Wheat Research Institution for granting us the opportunity to access and collect these valuable seeds for our research purposes.

## Conclusion

The future impact of climate change on wheat production is predicted to worsen due to increasing occurrences of severe droughts, heat stress, and other environmental stresses. Nevertheless, our research has indicated that the application of HRW to wheat seeds can significantly improve various growth factors such as germination rate, germination potential, vigor index, root-shoot length, number, and dry weight. It also enhances the accumulation of crucial components like RWC, chlorophyll content, and soluble proteins. Additionally, the use of HRW can increase the levels of essential nutrients such as Zn and Fe and boost the activity of antioxidant enzymes like CAT, SOD, and APX. This can mitigate oxidative damage and reduce ROS production in plants. The encouraging potential of HRW treatment in increasing nutrient and antioxidant levels in plants can lead to the development of new methodologies that can help adapt to the negative impacts of climate change on wheat production. Advanced molecular evidence indicates that HRW has a robust capacity to trigger the expression of the *Dreb1* gene, which is crucial in conferring drought tolerance to wheat plants. Still, more research is necessary to gain a deeper understanding of the topic, and to clarify the methodology for protecting against potential negative impacts.

### Supplementary Information

Supplementary Information.

## Data Availability

All data generated or analyzed during this study are included in this published article.
