# Supplementary material for: Hydrogen-rich water: a key player in boosting wheat (Triticum aestivum L.) seedling growth and drought resilience
Source: Sci Rep. 2023 Dec 18;13:22521. doi: 10.1038/s41598-023-49973-7 (PMC10728117; doi:10.1038/s41598-023-49973-7)
Supplement: Supplementary file 1 — Supplementary Information. [file 41598_2023_49973_MOESM1_ESM.docx]

**Hydrogen-Rich Water: A Key Player in Boosting Wheat (*Triticum aestivum* L.) Seedling Growth and Drought Resilience**

Md. Ariful Islam^1^, Most. Nourin Akther Shorna^2^, Shirmin Islam^1^, Suvro Biswas^1^, Jui Biswas^1^, Synthia Islam^3^, Amit Kumar Dutta^4^, Md. Salah Uddin^1^, Shahriar Zaman^1^, Md. Akhtar-E-Ekram^1^, Asad Syed^5^, Ling Shing Wong^6^, Md Sayeedul Islam^7,*^, Md. Abu Saleh^1,*^

^1^Microbiology Laboratory, Department of Genetic Engineering and Biotechnology, University of Rajshahi, Rajshahi-6205, Bangladesh

^2^Department of Botany, University of Rajshahi, Rajshahi-6205, Bangladesh

^3^Department of Agribusiness, Bangabandhu Sheikh Mujibur Rahman Agricultural University, Gazipur-1706, Bangladesh

^4^Department of Microbiology, University of Rajshahi, Rajshahi-6205, Bangladesh

^5^Department of Botany and Microbiology, College of Science, King Saud University, P.O. Box 2455, Riyadh, 11451, Saudi Arabia

^6^Faculty of Life and Health Sciences, INTI international University, Putra Nilai, 71800 Nilai, Negeri Sembilan, Malaysia

^7^Department of Biological Sciences, Graduate School of Science, Osaka University, Machikaneyama‑Cho 1‑1, Toyonaka, Osaka 560‑0043, Japan

^*^Correspondence to: Md. Abu Saleh (saleh@ru.ac.bd)

Md Sayeedul Islam ([islam.md.sayeedul.k2v@osaka-u.ac.jp](mailto:islam.md.sayeedul.k2v@osaka-u.ac.jp))

**Table S1:** Global and pairwise analysis of similarities (ANOSIM) showing significant dissimilarities in the HRW treated seeds at different time intervals compare to controls. *P*< 0.05 indicates significantly different global and/or pairwise ANOSIM tests. Significance levels: 0 ‘***’ 0.001 ‘**’ 0.01 ‘*’ 0.05, ‘.’ 0.1.

| Categories | ANOSIM | | |
| --- | --- | --- | --- |
|  | SQRT+Bray-Curtis | | |
| Treatments | R | P | Sign. Level |
| Global test | 0.921 | 0.001 | *** |
| Controls × 2h | 1 | 0.1 | . |
| Controls × 3h | 1 | 0.1 | . |
| Controls × 4h | 1 | 0.1 | . |
| Controls × 5h | 1 | 0.1 | . |
| Controls × 6h | 1 | 0.1 | . |
| 2h × 3h | 0.815 | 0.1 | . |
| 2h × 4h | 1 | 0.1 | . |
| 2h × 5h | 1 | 0.1 | . |
| 2h × 6h | 1 | 0.1 | . |
| 3h × 4h | 0.778 | 0.1 | . |
| 3h × 5h | 0.741 | 0.1 | . |
| 3h × 6h | 1 | 0.1 | . |
| 4h × 5h | 0.63 | 0.1 | . |
| 4h × 6h | 1 | 0.1 | . |
| 5h × 6h | 1 | 0.1 | . |

**Fig. S1:** Fig. Principal Coordinates Analysis (PCO) plot shows the ordination of treatment groups compare to control group. PCO based on the Bray-Curtis distances derived from the HRW treated wheat seeds (for 2h, 3h,4 h,5h, 6h treatment) and controls (without treatment)of 34 parameters of wheat. Variance explained for PCO1= 47.8% and variance explained PCO2= 27.2%.
